# El laboratorio en el diagnóstico multidisciplinar del desarrollo sexual anómalo o diferente (DSD): I) Fisiología, clasificación, abordaje y metodología II) Marcadores bioquímicos y genéticos diagnósticos en los 46,XX

**DOI:** 10.1515/almed-2020-0119

**Published:** 2021-05-24

**Authors:** Maria Luisa Granada, Laura Audí

**Affiliations:** Department of Clinical Biochemistry, Hospital Germans Trias i Pujol, Autonomous University of Barcelona, Badalona, España; Growth and Development Research Group, Vall d’Hebron Research Institute (VHIR), Center for Biomedical Research on Rare Diseases (CIBERER), Instituto de Salud Carlos III, Barcelona, Catalonia, España

**Keywords:** desarrollo sexual anómalo o diferente (DSD), diagnóstico bioquímico, diagnóstico genético, DSD 46,XX

## Abstract

**Objetivos:**

El desarrollo de las características sexuales femeninas o masculinas acontece durante la vida fetal, determinándose el sexo genético, el gonadal y el sexo genital interno y externo (femenino o masculino). Cualquier discordancia en las etapas de diferenciación ocasiona un desarrollo sexual anómalo o diferente (DSD) que se clasifica según la composición de los cromosomas sexuales del cariotipo.

**Contenido:**

En este capítulo se abordan la fisiología de la determinación y el desarrollo de las características sexuales femeninas o masculinas durante la vida fetal, la clasificación general de los DSD y su estudio diagnóstico clínico, bioquímico y genético que debe ser multidisciplinar. Los estudios bioquímicos deben incluir, además de las determinaciones bioquímicas generales, análisis de hormonas esteroideas y peptídicas, en condiciones basales o en pruebas funcionales de estimulación. El estudio genético debe comenzar con la determinación del cariotipo al que seguirá un estudio molecular en los cariotipos 46,XX ó 46,XY, orientado a la caracterización de un gen candidato. Además, se expondrán de manera específica los marcadores bioquímicos y genéticos en los DSD 46,XX, que incluyen el desarrollo gonadal anómalo (disgenesias, ovotestes y testes), el exceso de andrógenos de origen fetal (el más frecuente), fetoplacentario o materno y las anomalías del desarrollo de los genitales internos.

**Perspectivas:**

El diagnóstico de un DSD requiere la contribución de un equipo multidisciplinar coordinado por un clínico y que incluya los servicios de bioquímica y genética clínica y molecular, un servicio de radiología e imagen y un servicio de anatomía patológica.

## I. Fisiología, clasificación, abordaje y metodología

### 1) Fisiología de la diferenciación sexual y clasificación de sus variaciones

La determinación y el desarrollo de las características sexuales femeninas y masculinas durante la vida fetal constituyen procesos biológicos complejos que incluyen la expresión de cascadas de genes cuyas proteínas ejercen funciones altamente específicas, tanto en su localización como en su cronología [1], [2], [3]. El sexo genético se establece en el momento de la fertilización al fusionarse un espermatozoide (con un cromosoma sexual X o Y), con un ovocito (cromosoma X), dando lugar a una célula diploide 46,XX o 46,XY que determina el sexo genético. Durante las primeras semanas del embrión, el desarrollo de las estructuras gonadales y genitales es común a ambos sexos. El desarrollo del seno urogenital y del primordio adreno-gonadal acontece hacia la 4^a^ semana. El desarrollo de la gónada primitiva bipotencial requiere la expresión de una cascada de genes (entre ellos bien caracterizados en humanos: *EMX2, CBX2, NR5A1, GATA4, WT1*) [1], [3].

**Figura 1:**
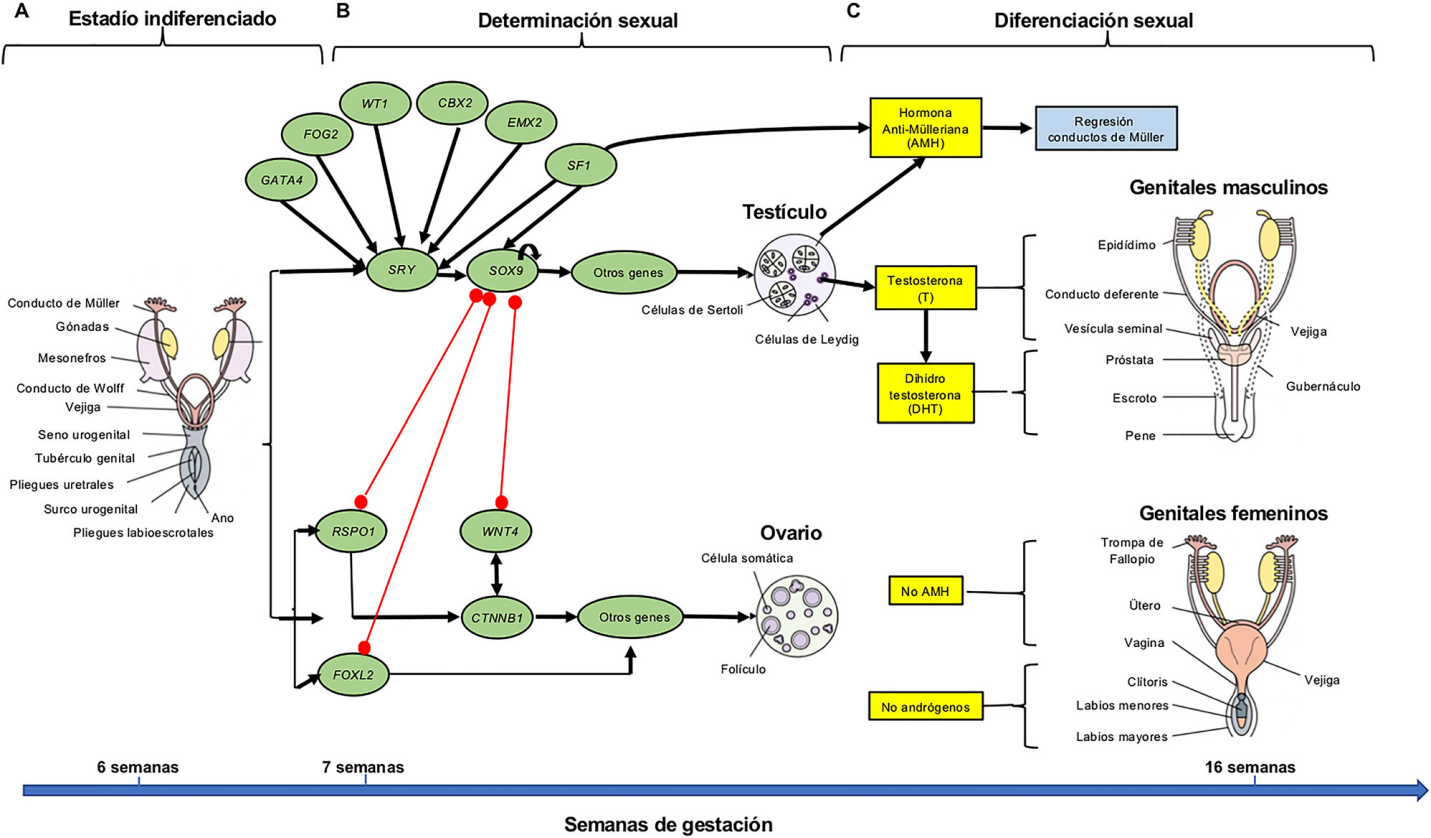
Desarrollo sexual humano durante la vida fetal. (A) Estadío indiferenciado: Las gónadas bipotenciales están desarrolladas a las 5 semanas, así como dos pares de conductos internos (conductos de Múller y de Wolff), los genitales externos constan del tubérculo genital, los pliegues uretrales que flanquean el surco urogenital, y los pliegues labioescrotales. Este estadío termina hacia la 6ª semana. (B) Determinación sexual: Comienza a desarrollarse entre las semanas 6ª y 7ª cuando las células somáticas y los gonocitos de las gónadas bipotenciales se diferencían en células testiculares u ováricas, dependiendo de la presencia y activación o represión de vías de señalización. Las flechas indican la activación de genes mientras que las líneas rojas indican represión de la expresión génica. (C) Diferenciación sexual: La diferenciación de genitales internos y externos depende de la presencia o ausencia de hormonas testiculares (hormona anti-Mülleriana [AMH], testosterona [T] y dihidrotestosterona [DHT]). Modificado de cita: León et al. [16].

A partir de la 6^a^ semana, la presencia de un cromosoma Y y la expresión de su gen *SRY* activa una cascada de genes que regulan el desarrollo de la gónada indiferenciada hacia un testículo [4] e inhibe la expresión de los genes que feminizan la gónada hacia un ovario [1, 3, 5, 6]. El desarrollo gonadal depende de complejas interacciones entre genes antagónicos que regulan procesos que finalmente determinan la formación de un testículo o de un ovario [7] (Figura 1).

En su etapa inicial indiferenciada, ambos sexos comparten un par de conductos genitales (conductos de Müller y conductos de Wolff) así como la morfología de los genitales externos (tubérculo genital y repliegues genitales) [8]. El desarrollo de genitales internos y externos en el sentido masculino depende de que el testículo secrete una serie de hormonas en cantidades adecuadas y siguiendo una cronología determinada. Los genitales internos masculinos requieren la secreción y acción de testosterona (T) que induce la diferenciación de los conductos de Wolff en epidídimo y conductos deferentes y de la hormona anti-Mülleriana (AMH) que actúa a través de su receptor (AMHR2) y provoca la regresión de los conductos de Müller [8]. El desarrollo de la próstata y de los genitales externos requiere el metabolismo local de la T que se transforma en dihidrotestosterona (DHT) por acción de la enzima 5-alfa-reductasa tipo 2 [9] (Figura 1).

En ausencia de AMH y de concentraciones elevadas de andrógenos (T y DHT) se desarrollan los genitales internos y externos femeninos (Figura 1). Aunque el modelo murino de ausencia de receptor de estrógenos (ER) puede sugerir que los estrógenos podrían actuar a través del receptor ER feminizando el tubérculo genital [10], la morfología de los genitales externos de una RN con resistencia completa al estradiol (E2) no ha sido descrita hasta la fecha.

La presencia de alteraciones en alguna de las etapas puede modificar el desarrollo sexual normal produciéndose “anomalías o diferencias en el desarrollo sexual” (DSD) (*“disorders or differences of sex development”*) [11], condiciones congénitas en las que el desarrollo de los sexos cromosómico, gonadal y/o genital es atípico o distinto al más frecuente.

Las causas de DSD se clasifican según el Consenso de Chicago [11] en función del cariotipo. Existen tres grupos principales: - 1) los DSD cromosómicos, cuando la composición de los cromosomas sexuales es distinta de los pares XX o XY; - 2) los DSD 46,XX con cariotipo femenino, - 3) los DSD 46,XY con cariotipo masculino (Tabla 1). Cada grupo se subdivide en distintos tipos (Tabla 1). En los Grupos 2 y 3, los genes implicados son muy numerosos y aumentan de año en año.

**Tabla 1:** Clasificación del desarrollo sexual anómalo o diferente (DSD) según los cromosomas sexuales presentes en el cariotipo [11].

1) DSD por cromosomas sexuales	
47,XXY: síndrome de Klinefelter y variantes 45,X0 y mosaicos 45,X0/46,XX (síndrome de Turner y variantes) 45,X0/46,XY mosaico (disgenesia gonadal mixta) 46,XX/46,XY mosaico (DSD ovotesticular) 47,XYY	
**2) DSD con cariotipo 46,XX**	
1. Desarrollo gonadal anómalo	Disgenesia gonadal parcial (DGP) o completa (DGC) DSD ovotesticular DSD testicular
2. Desarrollo genital anómalo por exceso de andrógenos	**Producción fetal:** Déficit de 21-hidroxilasa Déficit de 3β-hidroxiesteroide deshidrogenasa tipo 2 Déficit de 11-β-hidroxilasa Resistencia a glucocorticoides Resistencia a estrógenos
	**Producción feto-placentaria:** Déficit de P450 oxido-reductasa Déficit placentario y fetal de aromatasa Tumores fetales o placentarios productores de andrógenos
	**Origen materno:** Agentes terapéuticos o contaminantes medioambientales HSC materna Tumores maternos virilizantes (luteomas, tumor de Krukenberg)
3. Desarrollo anómalo de los genitales internos	Síndrome pie-mano-genital MURCS (Müllerian aplasia, Renal aplasia, Cervico-thoracic Somite abnormalities) MRKH (Mayer-Rokitansky-Küster-Hauser) syndrome, tipos I y II
**3) DSD con cariotipo 46,XY**	
1. Desarrollo gonadal anómalo	Disgenesia gonadal parcial (DGP) o completa (DGC) DSD ovotesticular DSD ovárico
2. Desarrollo genital anómalo por anomalías en la síntesis o en la acción de los andrógenos	**Anomalías en la síntesis de andrógenos:** Insensibilidad a la LH (aplasia/hipoplasia de células de Leydig) Déficit de 7-dehidrocolesterol reductasa (síndrome de Smith-Lemli-Opitz) Déficit de proteína StAR (hiperplasia suprarrenal congénita lipoidea) Déficit de colesterol desmolasa Déficit de 3β-hidroxi-esteroide deshidrogenasa tipo 2 Déficit de 17α-hidroxilasa/17–20 desmolasa Déficit de P450 oxidoreductasa Déficit de citocromo B5 Déficit en la esteroidogénesis de la vía trasera Déficit de 17β-hidroxi-esteroide deshidrogenasa tipo 3 Déficit de 5α-reductasa tipo 2 Hipospadias y/o criptorquidia aislados
	**Anomalías en la acción de los andrógenos:** Insensibilidad completa o parcial a los andrógenos Agentes terapéuticos o contaminantes medioambientales
3. Desarrollo genital anómalo por anomalías en la síntesis o en la acción de la hormona anti-Mülleriana (AMH)	**Persistencia de los conductos de Müller:** Déficit de hormona anti-Mülleriana Resistencia a la hormona anti-Mülleriana
4. Síndromes malformativos complejos	Síndromes malformativos con desarrollo genital anómalo (anomalías cloacales, síndrome de Aarskog, síndrome de Robinow, etc.) Crecimiento intrauterino retardado, precoz y severo

En el Grupo 1, los DSD cromosómicos quedan definidos por el número o la estructura de los cromosomas sexuales (Tabla 1). Los más frecuentes son el 47,XXY (síndrome de Klinefelter), el 45,X0 (síndrome de Turner) y sus variantes, incluido el mosaico 45,X/46,XY (o disgenesia gonadal mixta), el mosaico 46,XX/46,XY (verdadera quimera sexual cromosómica o DSD ovotesticular) y el 47,XYY.

En el Grupo 2, 46,XX, pueden existir (Tabla 1): - 1) Anomalías en el desarrollo gonadal (disgenesia gonadal completa [DGC], parcial [DGP], quimera ovotesticular [DSD ovotesticular] o desarrollo testicular [DSD testicular]); - 2) Desarrollo genital anómalo por exceso de andrógenos (de producción fetal, feto-placentaria o de origen materno) que virilizan los genitales externos; - 3) Desarrollo anómalo de los genitales internos.

En el Grupo 3, con cariotipo 46,XY pueden existir (Tabla 1): - 1) Anomalías en el desarrollo gonadal (DGC, DGP, desarrollo ovotesticular [DSD ovotesticular] o desarrollo ovárico [DSD ovárico]); - 2) Anomalías en la síntesis o en la acción de los andrógenos; - 3) Anomalías en la síntesis o en la acción de la AMH; - 4) Síndromes malformativos complejos que afectan el desarrollo genitourinario y digestivo y el crecimiento intrauterino retardado, precoz y severo que se acompaña de hipospadias.

Excepto para los DSD cromosómicos del Grupo 1 (sobre todo el síndrome de Klinefelter con cariotipo 47,XXY) y los niños con cariotipo 46,XY del Grupo 3 que nacen con hipospadias, la frecuencia poblacional para los DSD de los Grupos 2 y 3 es tan baja que entran dentro de la categoría de las llamadas “enfermedades raras” (ER) (frecuencia poblacional inferior a 1/2.000).

### 2) Equipos multidisciplinares para el diagnóstico de los DSD

El DSD puede manifestarse en el recién nacido (RN) o poco después, por la presencia de genitales externos ambiguos, por discordancia entre el cariotipo prenatal y el desarrollo genital, cuando existe una historia familiar de DSD, cuando se presenta una insuficiencia suprarrenal aguda o cuando se detecta la presencia de una gónada en una hernia inguinal. Posteriormente, durante el desarrollo puberal, por discordancias entre el desarrollo gonadal y el genital. Además, algunos adultos no diagnosticados pueden consultar por infertilidad o por otros problemas de salud como una hipertensión arterial y la investigación de las posibles etiologías conducir a la detección de algún DSD.

Diagnosticar la causa de un DSD puede ser más o menos complejo, dependerá, en parte, de los conocimientos y la habilidad de cada profesional involucrado así como de la funcionalidad del equipo multidisciplinar de especialistas [11], [12]. En todos los protocolos se insiste en que el diagnóstico médico de un DSD requiere la contribución de un equipo de especialistas que colaboren con un clínico coordinador [12], [13], y que incluye un servicio de bioquímica (bioquímica general y marcadores u hormonas específicas), un servicio de genética clínica y molecular (cariotipo inicial y la interpretación del resultado de otras exploraciones orientarán el desarrollo de otros análisis), un servicio de radiología e imagen (una ecografía pélvica para detectar las estructuras genitales internas y la presencia de gónadas intraabdominales) y un servicio de anatomía patológica (cuando se requiere el análisis de la estructura de las gónadas).

Se han elaborado y propuesto muchos algoritmos diagnósticos [13], [14], [15], [16] que han ido evolucionando en función de las tecnologías asequibles, principalmente en los campos de la imagen y la bioquímica, pero sobre todo del diagnóstico molecular [17], [18], [19], [20], [21].

### 3) Exploraciones bioquímicas y genéticas en el diagnóstico de los DSD

#### a) Exploraciones bioquímicas basales

Las pruebas bioquímicas y, en especial, las magnitudes hormonales juegan un papel muy importante tanto en el diagnóstico inicial de los DSD como en el control evolutivo y la monitorización del tratamiento. Podemos diferenciar dos grandes grupos de hormonas: las hormonas esteroideas y las peptídicas.

**Figura 2:**
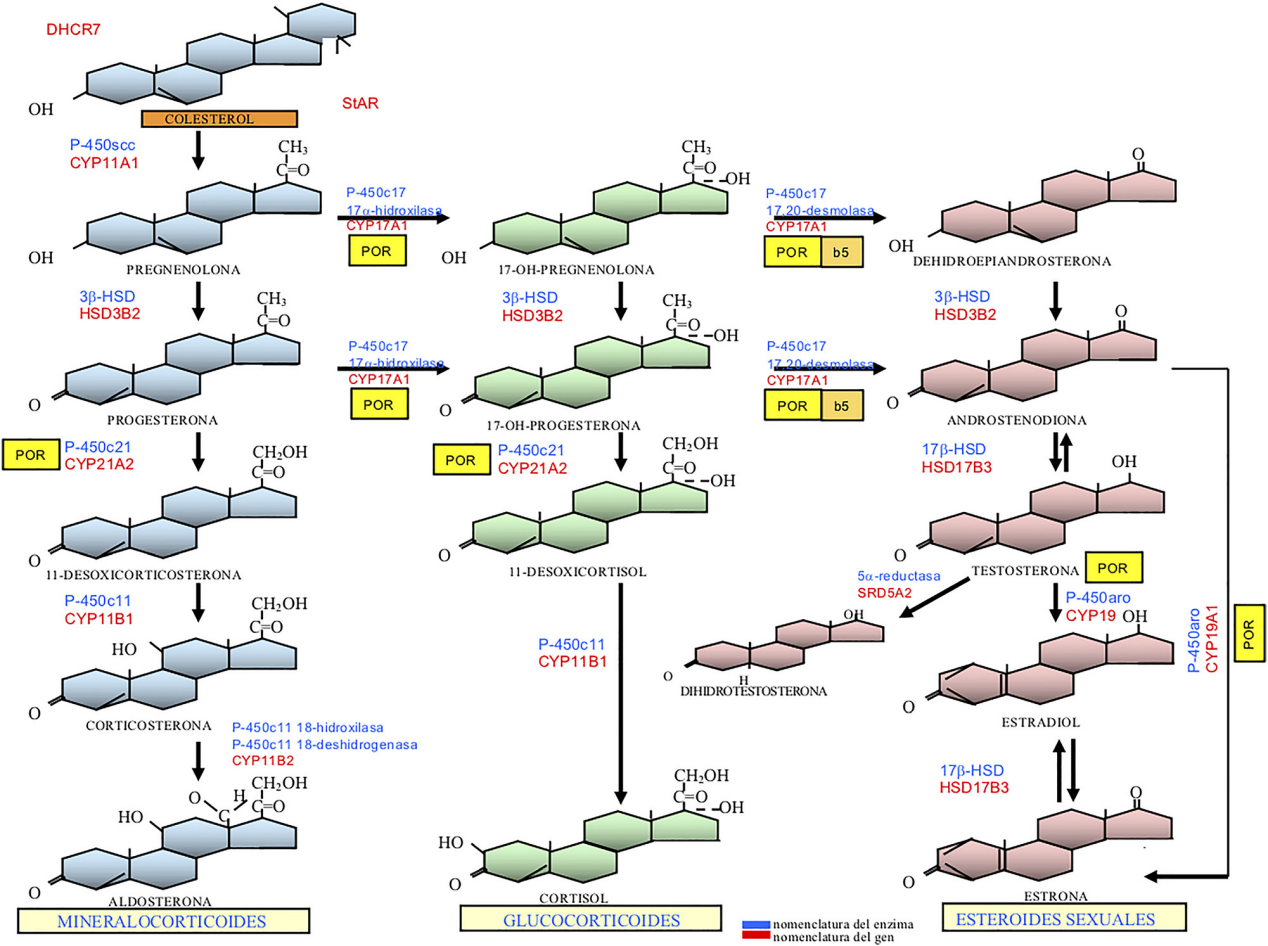
Esteroidogénesis suprarrenal y gonadal. Desde el colesterol, la biosíntesis progresa en la glándula suprarrenal hasta el cortisol (vía de los glucocorticoides) y hasta la aldosterona (vía de los mineralocorticoides). En las gónadas los precursores progresan hacia los esteroides sexuales: testosterona (T) como principal andrógeno y estradiol como principal estrógeno. T es transformada periféricamente en dihidrotestosterona (DHT) como andrógeno más potente. En azul: abreviación de los enzimas; en rojo: abreviación de los genes que codifican cada enzima; cuadro amarillo: coenzima POR (P450-oxidoreductasa); cuadro beis: citocromo b5. DHCR7 = gen DHCR7 (7alfa-dehidrocolesterol reductasa) StAR = steroid acute regulatory protein (gen StAR) P-450scc = P-450 side-chain cleavage = colesterol-desmolasa (gen CYP11A1) 3β-HSD = 3β-hidroxiesteroide-deshidrogenasa tipo 2 (gen HSD3B2) P-450c17 = 17α-hidroxilasa/17,20-desmolasa o liasa (gen CYP17A1) P-450c21 = 21-hidroxilasa (gen CYP21A2) P-450c11 = 11β-hidroxilasa tipo 1 (gen CYP11B1) P-450c11 18-hidroxilasa (corticosterona metil-oxidasa tipo 1 [CMO-I]) y P-450c11 18-deshidrogenasa (corticosterona metil-oxidasa tipo II [CMO-II]) (gen CYP11B2) 17β-HSD = 17-hidroxiesteroide deshidrogenasa tipo 3 ó 17-cetorreductasa (gen HSD17B3) 5α-reductasa tipo 2 (gen SRD5A2) P-450aro = aromatasa (gen CYP19A1).

Las hormonas esteroideas (Figura 2) se sintetizan a partir del colesterol en el córtex adrenal, las gónadas y la placenta aunque se metabolizan en numerosos tejidos periféricos. Su medición en sangre y en orina ha ido evolucionando hasta las técnicas actuales de inmunoensayo y de espectrometría de masas. Disponemos de inmunoensayos comerciales para los esteroides más solicitados en la práctica clínica; sin embargo, para algunos parámetros de interés en el diagnóstico de los DSD como la corticosterona, la desoxicorticosterona, la 17-OH-pregnenolona y la DHT se deberá recurrir a cromatografía tándem líquida-espectrometría de masas (LC-MS/MS) [22], [23]. Las sociedades científicas internacionales recomiendan la utilización de métodos basados en la espectrometría de masas (LC-MS/MS y GC-MS/MS) para la medida de esteroides sexuales y sus precursores, en el diagnóstico de los DSD, en especial, en los neonatos [24]. Esta metodología permite determinar en una misma muestra perfiles de esteroides, incluyendo metabolitos para los que no se dispone de inmunoensayos específicos [25]. Los esteroides se pueden medir en diferentes matrices: suero, sangre, saliva, orina [26]. Es muy importante que el laboratorio trabaje según estándares de calidad, que participe en programas externos de calidad y que disponga de intervalos de referencia específicos de edad y sexo [24]. Los esteroides adrenales tienen un marcado ritmo de secreción circadiano por lo que se aconseja realizar las determinaciones en muestras obtenidas a primera hora de la mañana (8–9 a.m.).

La medición de hormonas proteicas se realiza fundamentalmente por inmunoensayos no competitivos que tienen gran sensibilidad, pero cuya especificidad no siempre es conocida. La falta de métodos de referencia y las diferencias en la estandarización se traducen en una falta de comparabilidad entre ensayos haciendo indispensable disponer de valores de referencia específicos para los diferentes grupos de edad y sexo para cada ensayo [27], [28]. Las hormonas peptídicas relacionadas con el desarrollo sexual presentan un marcado dimorfismo sexual. En los primeros años de vida en el sexo masculino las concentraciones de LH son más elevadas que las de FSH y el cociente LH/FSH es claramente más elevado [29]. Las concentraciones de AMH son 100 veces más elevadas en el varón que en la mujer y las de INHB 10 veces superiores. Recientemente se ha publicado una guía para la determinación de hormonas peptídicas en el estudio de los DSD [30].

#### b) Pruebas funcionales

En algunos casos la medición de hormonas basales no es suficientemente informativa y es necesario realizar pruebas de estimulación para poner en evidencia déficits de secreción.

Se realizan fundamentalmente tres tipos de pruebas funcionales:

##### b-1) Prueba de estimulación con Corticotropina (ACTH) [31]

Su objetivo es el estudio de la esteroidogénesis adrenal (Figura 2). Se administra por vía endovenosa ACTH sintético (1–24) (Cosyntropin o Synacthen®) a dosis de 0,25 mg (en bebés se puede reducir a 0,125 mg). Se determinan diferentes hormonas y precursores basalmente y a los 60 minutos del estímulo para valorar déficits enzimáticos adrenales [32].

##### b-2) Prueba de estimulación con gonadotropina coriónica (HCG) [33]

La HCG estimula la producción de andrógenos testiculares al unirse al receptor de LH-HCG de las células de Leydig. Existen diferentes protocolos de estimulación. La medida de andrógenos y sus precursores se realiza antes y a las 48–72 horas de la última inyección. En el diagnóstico de DSD es importante medir T, sus precursores y su metabolito DHT para diagnosticar déficits enzimáticos en la esteroidogénesis testicular y periférica (Figura 2).

##### b-3) Prueba de estimulación con LHRH o sus análogos

Se puede realizar administrando el factor hipotalámico LHRH, liberador de LH y FSH, **(**Prueba de Luforan® 100 µg i.v y en niños de 25 a 50 µg) o más recientemente, utilizando análogos del LHRH (acetato de leuprorelina [Procrin®], acetato de buserelina). Una respuesta de LH superior a 5 UI/L se considera indicativo de activación central del eje hipotálamo-hipófiso-gonadal (HHG) [34], [35], [36].

#### c) Exploraciones genéticas

##### c-1) Citogenética y cariotipo

El cariotipo es la base para la clasificación de un DSD en alguno de los tres grupos diagnósticos que depende de los cromosomas sexuales presentes (Tabla 1). La técnica clásica es de citogenética, aunque recientemente se están utilizando técnicas de hibridación de alta resolución [21].

Además de anomalías en los cromosomas sexuales, algunos DSD pueden ser portadores de variaciones en el número de copias (VNC) (deleciones, duplicaciones, translocaciones), tanto en autosomas como en cromosomas sexuales; esto es especialmente importante cuando el fenotipo incluye otras anomalías adicionales al DSD [37], [38], [39], [40]. Las VNC se detectan mediante técnicas de hibridación de alta resolución (*array-CGH: array-complementary genome hybridisation*) y podrán ser detectadas al analizar el cariotipo mediante técnicas de array-CGH.

##### c-2) Análisis de genes

Las causas monogénicas de DSD más frecuentes se fueron describiendo a lo largo del último tercio del siglo XX cuando se fueron clonando genes que codifican proteínas cuya alteración era conocida por los fenotipos clínico y bioquímico. Esto es especialmente cierto para los déficits enzimáticos de la esteroidogénesis adrenal y gonadal (Figura 2), tanto en los DSD 46,XX como en los 46,XY, y en el síndrome de insensibilidad completa a los andrógenos. En cambio, los genes implicados en la determinación y el desarrollo de las gónadas masculina y femenina van siendo progresivamente detectados a partir de estudios familiares, modelos animales y estudios funcionales *in vitro* [7], [41]. Un buen número de genes relacionados con el desarrollo de un DSD codifican factores de transcripción reguladores de otros genes (por ejemplo: *AR, DAX1, DMRT1, FOXL2, NR5A1, SOX3, SOX9* y *SRY*) y, habiéndose demostrado la existencia de mutaciones en regiones no-codificantes del genoma, se puede anticipar que la exploración del genoma no-codificante permitirá la progresiva comprensión de la causa de algunos DSD que carecen de diagnóstico molecular [42].

El análisis estructural de un gen candidato individual se realiza mediante secuenciación automática de Sanger que comporta la amplificación mediante PCR de las regiones codificantes y flanqueantes y, eventualmente, de la región promotora. Sin embargo, la introducción de técnicas secuenciación de alto rendimiento permite actualmente el análisis del exoma completo (regiones codificantes) o seleccionar el análisis de un panel de genes candidatos. Además, la amplia expresividad en los fenotipos de los DSD podría explicarse en algunos casos por una afectación oligogénica en la que la interacción de varios genes relacionados puede ser responsable de un fenotipo único para cada individuo [43]. La mejora progresiva de la calidad de estas técnicas y de su precio, las han hecho accesibles a la mayor parte de laboratorios de diagnóstico molecular, de modo que el análisis de un gen individual o de una de sus regiones quedará progresivamente limitado al diagnóstico de un nuevo paciente familiar de un caso bien caracterizado [21, 44–49].

## II. Marcadores bioquímicos y genéticos en los DSD 46,XX

La Tabla 2

**Tabla 2:** Diagnósticos clínicos y genes involucrados en el desarrollo sexual anómalo o diferente (DSD) de causa monogénica.

DSD con cariotipo 46,XX		
Diagnóstico clínico (from clinico untilö adicional)	Gen (locus)	OMIM (herencia) (fenotipo adicional)
1. DSD 46,XX por anomalías en el desarrollo gonadal: disgenesia gonadal, DSD ovotesticular, DSD testicular		
Disgenesia gonadal	*BMP15* (Xp11.22)	300510/300247 (D)
Disgenesia gonadal	*ESR2* (14q23.2-q23.3)	618187 (AD)
DSD testicular	*FGF9* (13q12.11)	600921 (AD:dup) (Un solo caso descrito)
Disgenesia gonadal	*FOXL2* (3q22.3)	608996 (AD) (Blefarofimosis, epicanto inverso y ptosis, tipos I y II)
Disgenesia gonadal	*MYRF (11q12.2)*	608329 (AD) 618280 (AD) (Síndrome cardíaco urogenital)
DSD testicular	*NR2F2 (15q26.2*	615779 (AD) (cardiopatía congénita, hernia diafragmática, síndrome blefarofimosis-ptosis-epicanto inverso)
1) Disgenesia gonadal 2) DSD ovotesticular 3) DSD testicular	1) *NR5A1* (9q33.3) 2) *NR5A1*(9q33.3)(p.Arg92Trp) 3) *NR5A1* (p.Arg92Trp)	612964 (AD) 617480 (AD)
Disgenesia gonadal	*NUP107* (12q15)	607617 (AR) (descrito en familia consanguínea; otros fenotipos con síndrome nefrótico)
DSD ovotesticular	*RSPO1* (1p34.3)	610644 (AR) (Hiperqueratosis palmoplantar, carcinoma cutáneo de células escamosas)
1) DSD ovotesticular 2) DSD testicular	*SOX3* (Xq27.1)	313430 (XL:dup)
Disgenesia gonadal	*SOX8* (16p13.3)	605923 (Insuficiencia ovárica primaria)
1) DSD ovotesticular 2) DSD testicular	*SOX9* (17q24.3)	278850 (AD:dup)
1) DSD ovotesticular 2) DSD testicular	*SOX10* (22q13.1)	609136 (AD:dup) (síndromes de Waardenberg y de Hirschsprung, neuropatía periférica)
1) DSD ovotesticular 2) DSD testicular	*SRY* (Yp11.2)	400045 (T)
1) DSD ovotesticular 2) DSD testicular	*WNT4* (1p36.12)	158330 (AD) 611812 (AR): síndrome SERKAL (Sex Reversal dysgenesis of Kidneys, Adrenals and Lung), bialélico letal
1) DSD ovotesticular 2) DSD testicular	*WT1* (11p.13)	AD

**2. DSD 46,XX con desarrollo gonadal normal pero genital anómalo por exceso de andrógenos fetales o fetoplacentarios**		

HSC por déficit de 21-hidroxilasa	*CYP21A2* (6p21.33)	201910 (AR) (Déficit suprarrenal)
HSC por deficit de 3α-hidroxi-esteroide deshidrogenasa tipo 2	*HSD3B2* (1p12)	201810 (AR) (Déficit suprarrenal y gonadal)
HSC por déficit de 11β-hidroxilasa	*CYP11B1* (8q24.3)	202010 (AR) (Déficit suprarrenal)
Insensibilidad a los glucocorticoides	*GRα (NR3C1)* 5q31.3	615962 (AD) (Hipertensión)
Insensibilidad a los estrógenos	*ESR1* (6q25.1-q25.2)	615363 (AR) (Hipercrecimiento, osteoporosis, ovario poliquístico) (Un solo caso descrito)
Déficit de P450-oxidoreductasa	*POR* (7q11.23)	201750 (AR) (déficit de 17α-hidroxilasa, 21-hidroxilasa y aromatasa variables) (síndrome de Antley-Bixler syndrome, ± craniosinostosis)
Déficit de aromatasa	*CYP19A1* (15q21.2)	613546 (AR) (virilización materna y fetal)

**3. DSD 46,XX con desarrollo gonadal normal pero anómalo de los conductos Müllerianos**		

Síndrome Pie-Mano-Genital	*HOXA13* (7p15.2)	140000 (AD)
Síndrome MURCS (Müllerian Aplasia, Renal aplasia, Cervico-thoracic Somite abnormalities) Síndrome MRKH (Mayer-Rokitansky-Küster-Hauser), tipos I y II Aplasia Mülleriana e hiperandrogenismo	Multigénico: Del 17q12 CNV en 17q12, 1q21.1, 22q11.21, Xq21.31 Dupl *SHOX* *WNT4* (1p36.12)	601076 277000 614527/267400/192050 158330 (AD)

DSD, desarrollo sexual anómalo o diferente; DGP, disgenesia gonadal parcial; DGC, disgenesia gonadal completa; HSC, hiperplasia suprarrenal congénita; D, dominante; AD, autosómico dominante; AR, autosómico recesivo; XL, ligado al X; T, translocación; Dup, duplicación; Del, deleción; ¿?, desconocido; CNV, variación en número de copias.

expone la lista de causas monogénicas de DSD en el Grupo con cariotipo 46,XX. La lista se va alargando de año en año, sobre todo entre las causas de desarrollo gonadal disgenético.

### 1) Desarrollo gonadal anómalo

Las anomalías en el desarrollo gonadal con cariotipo 46,XX comprenden las disgenesias gonadales parciales y completas (DGP y DGC), las gónadas ovotesticulares (DSD ovotesticular) y el desarrollo de testículo (DSD testicular) (Tabla 1).

Las más frecuentes son las DGP y DGC. Ninguna de las dos da lugar a ambigüedad genital, siendo el fenotipo al nacer femenino. Suelen manifestarse clínicamente en la pubertad, cuando ésta se retarda y/o no se desarrolla. Los marcadores bioquímicos muestran aumento de la LH y FSH, AMH indetectable y estradiol (E2) a concentraciones prepuberales. Otros precursores como la androstendiona, la 17-hidroxiprogesterona (17OH-P) y la T también tendrán concentraciones prepuberales, mientras que la dehidroepiandrosterona (DHEA) y su sulfato habrán aumentado durante la adrenarquia normal. Una forma clínica más leve y relativamente frecuente es la menopausia precoz o fallo ovárico prematuro, siendo sus marcadores bioquímicos el aumento precoz de LH y FSH, una disminución de la AMH (buen marcador de la reserva ovárica de folículos), la disminución de E2 y progesterona (P). Se van describiendo causas monogénicas (Tabla 2), entre ellas mutaciones inactivadoras en los genes *BMP15, ESR2, FOXL2, MYRF, NR5A1, NUP107* y *SOX8.* En la mayoría de casos el efecto es dominante (excepto *NUP107*) y en algunos casos se asocian otras características fenotípicas (Tabla 2).

El desarrollo ovotesticular o testicular presenta ambigüedad genital (incluso genitales externos completamente masculinos) desde el nacimiento, ya que ha existido exposición a concentraciones elevadas de T durante el desarrollo fetal. Los marcadores bioquímicos en el RN o lactante serán similares a los hallados en el sexo masculino (46,XY); durante la infancia la capacidad de secreción de T por las gónadas se podrá evaluar mediante el test de HCG. En la pubertad se produce un aumento de T, que no alcanza niveles masculinos normales por lo que se acompaña de un aumento de LH y FSH. La mayoría de causas monogénicas pueden dar lugar tanto a DSD ovotesticular como testicular (Tabla 2). La primera conocida fue la translocación de un fragmento del cromosoma Y que contenga el gen *SRY* sobre un autosoma. Algunos se asocian a fenotipos complejos como las mutaciones en *NR2F2, RSPO1, SOX10, WNT4* y recientemente *WT1*; en el caso del gen *NR5A1*, sólo la mutación p.Arg92Trp provoca el desarrollo ovotesticular o testicular; también se describen duplicaciones en los genes *FGF9, SOX3* y *SOX9*.

### 2) Desarrollo genital anómalo por exceso de andrógenos

Cuando el desarrollo gonadal es ovárico y el de los genitales internos es femenino, el exceso de andrógenos durante el desarrollo fetal produce la virilización de los genitales externos. El origen de los andrógenos puede ser el propio feto, la unidad fetoplacentaria o la madre (Tabla 1).

#### a) Aumento de andrógenos de origen fetal

En la mayoría de los RN con cariotipo 46,XX que presentan virilización de los genitales externos, ésta es debida a una hiperplasia suprarrenal congénita (HSC). La causa más frecuente es el déficit de 21-hidroxilasa [32], [50] (gen *CYP21A2*), que en su forma “simple virilizante” presenta un déficit de cortisol, aumento de ACTH, acúmulo del precursor 17OH-P, de androstendiona y de T (Figura 2). En las formas más graves, o “perdedoras de sal”, se asocia un déficit de aldosterona con hiponatremia, hiperkaliemia y aumento de la actividad de la renina plasmática (ARP). El diagnóstico bioquímico se realiza ante el hallazgo de concentraciones elevadas de 17OH-P (basal o post ACTH > 300 nmol/L [>10.000 ng/dL]), androstendiona y T. También es necesario medir glucosa y electrolitos en sangre y la ARP [51]. Es importante tener valores de referencia estratificados por edad gestacional ya que en el RN prematuro las concentraciones de 17OH-P son mucho más elevadas, siendo causa de diagnósticos falsamente positivos [52]. La determinación del 21-deoxicortisol en suero, producido por la 11β-hidroxilación de la 17OH-P, puede ser útil para minimizar los diagnósticos falsos positivos ya que se eleva en el déficit de 21-hidroxilasa pero no en otros déficits adrenales ni en los RN prematuros, aunque es una determinación que no está disponible en muchos laboratorios clínicos [53].

**Figura 3:**
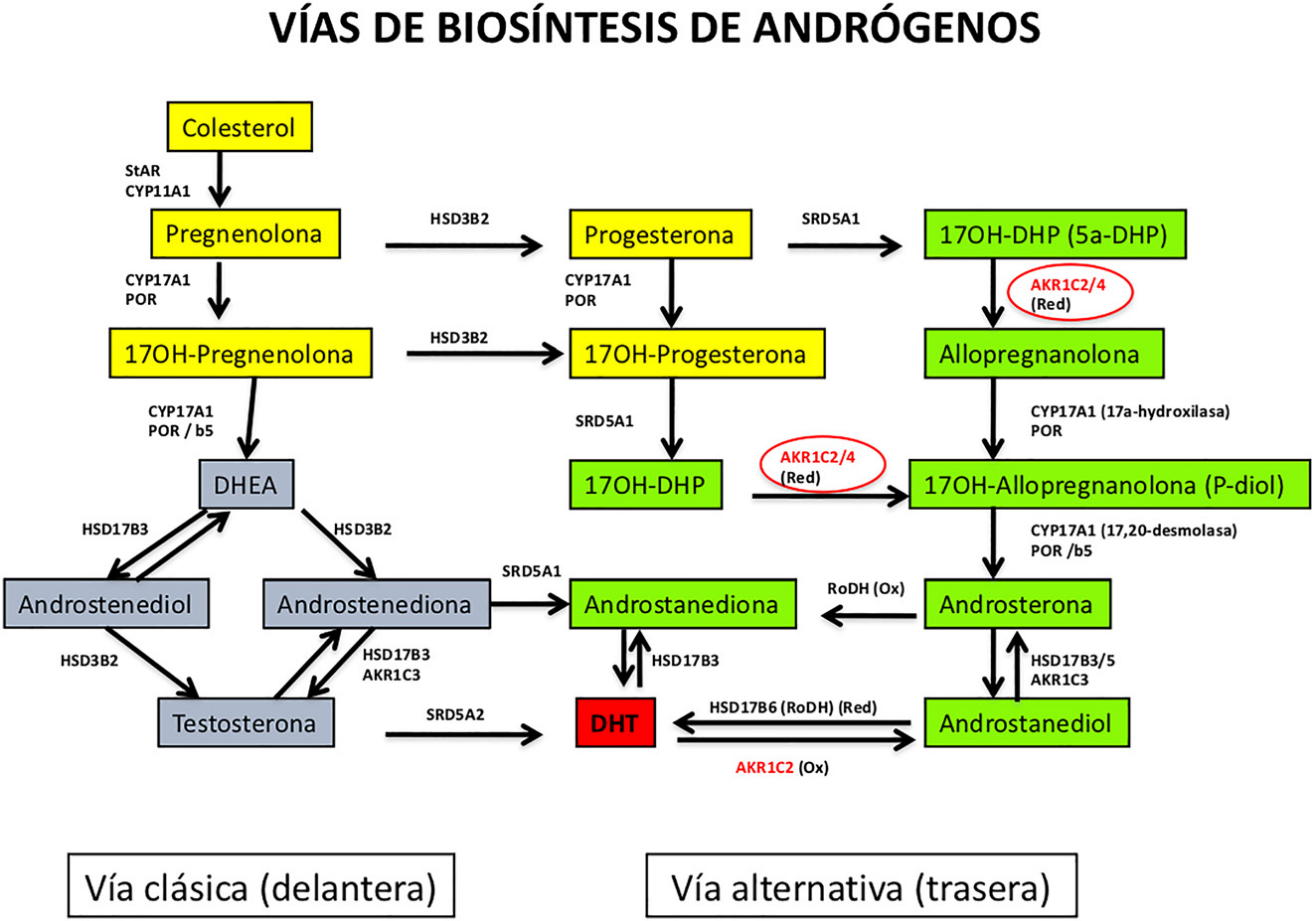
Vías de biosíntesis de andrógenos: vía clásica (o delantera) y vía alternativa (o trasera). La vía clásica progresa desde el colesterol hasta la testosterona (T), pasando por la pregnenolona (intervienen la proteína StAR [gen STAR] y el enzima colesterol desmolasa [gen CYP11A1]), la 17-OH-pregnenolona (enzima 17α-hidroxilasa [gen CYP17A1] y coenzima P450-oxidoreductasa [gen POR]), la dehidroepiandrosterona (DHEA) (enzima 17,20-desmolasa [gen CYP17A1], coenzima P450-oxidoreductasa [gen POR] y citocromo b5 [gen CYB5A]), la androstenediona o el androstenediol (enzimas 3β-hidroxiesteroide deshidrogenasa tipo 2 [gen HSD3B2], 17beta-hidroxiesteroide deshidrogenasa tipo 3 [gen HSD17B3] y aldo-keto reductasa familia 1 miembro C3 [gen AKR1C3]). La T es transformada en dihidrotestosterona (DHT) mediante el enzima 5α-reductasa tipo 2 (gen SRD5A2). También la DHT puede proceder de la transformación de androstenediona en androstanediona (enzima 5α-reductasa tipo 1 [gen SRD5A1]) y de ésta en DHT (enzima 17β-hidroxiesteroide deshidrogenasa tipo 3 [gen HSD17B3]). La vía alternativa llega a la síntesis de DHT sin pasar por la T. En ella, la progesterona (procede de la pregnenolona) y la 17OH-progesterona (procede de la progesterona) son transformadas en 17OH-DHP (5α-dihidroxi-progesterona). Ésta llega a androsterona la cual pasa a androstanediona o androstanediol, las cuales pasan a DHT (intervienen varios enzimas previamente descritos así como aldo-keto reductasa familia 1 miembros C2 y C4 [genes AKR1C2 y AKR1C4], retinol-dehidrogenasa [gen RODH], 17β-hidroxiesteroide deshidrogenasa tipo 5 y tipo 6 [genes HSD17B5 y HSD17B6]). (Ox) = oxidación; (Red) = reducción.

La medida de esteroides urinarios mediante GC-MS/MS muestra la activación de la vía alternativa de la puerta trasera (*alternative backdoor pathway*) (Figura 3), con incremento del 5α-pregnane-3α,17α-diol-20-one (P-diol), pregnanetriol (P-triol), la 17OH-pregnanolona y aumento del cociente androsterona/etiocolanolona [54].

Existen formas leves del déficit enzimático, llamadas “no clásicas” o tardías, que se manifiestan por una pubarquia precoz con discreta aceleración de la velocidad de crecimiento y de la maduración ósea, y aparición de vello pubiano. Bioquímicamente existe un discreto aumento de la 17OH-P basal con aumento o no de la androstendiona y de la T. La estimulación con ACTH demostrará el exceso de 17OH-P (31–300 nmol/L; 1.000–10.000 ng/dL).

El diagnóstico molecular deberá confirmar la presencia de mutaciones en el gen *CYP21A2,* en homocigosis o en heterocigosis compuesta, cuyo efecto es la anulación completa o casi completa de la actividad enzimática en las formas severas, tanto con pérdida salina como en la virilizante simple. Existe una asociación entre el genotipo y el grado de virilización de las pacientes con formas clásicas del déficit enzimático [55]. En las formas “no clásicas” uno de los alelos debe ser portador de una mutación de efecto leve, que permita la síntesis de una cierta actividad enzimática. Las mutaciones en el gen *CYP21A2* constituyen la anomalía molecular más frecuente en humanos, aunque su incidencia varía según las zonas geográficas y las estructuras sociales [32], [56] (Tabla 2).

Otras causas mucho menos frecuentes de HSC son el déficit de 3β-hidroxiesteroide deshidrogenasa tipo 2 (3βHSD2), el de 11β-hidroxilasa y el déficit de citocromo P-450 oxidorreductasa (POR) [57].

El enzima 3βHSD2 (gen *HSD3B2*) cataliza dos reacciones secuenciales, transformando la pregnenolona en P, la 17OH-pregnenolona en 17OH-P y la DHEA en androstendiona (Figura 2). Las pacientes con déficit grave presentan HSC por déficit en la síntesis de cortisol y de aldosterona. Existe un aumento de los esteroides Δ5 (pregnenolona, 17OH-pregnenolona, DHEA) y de la relación de éstos con los esteroides Δ4 (P, 17OH-P y androstendiona). Sin embargo, las concentraciones de 17OH-P, pueden estar aumentadas por la conversión periférica de Δ5-17OH-pregnenolona por la enzima 3βHSD tipo 1. El diagnóstico bioquímico se basa en la demostración de concentraciones elevadas de 17OH-pregnenolona (>150 nmol/L) basal o tras estimulación con ACTH [58]. El diagnóstico molecular deberá confirmar la presencia en homocigosis o heterocigosis compuesta de mutaciones inactivadoras en el gen *HSD3B2* (Tabla 2).

El enzima 11β-hidroxilasa convierte el 11-desoxicortisol en cortisol y la 11-desoxicorticosterona en corticosterona (Figura 2). Su déficit da lugar a una HSC con déficit de cortisol y de aldosterona que deriva a la síntesis de andrógenos (T) y se asocia a una virilización muy importante del feto femenino. El perfil hormonal se caracteriza por disminución de cortisol y de aldosterona, con aumento de ACTH pero ARP inhibida. El indicador diagnóstico más robusto es el aumento de 11-desoxicortisol y de 11-desoxicorticosterona, aunque estos parámetros no están disponibles en muchos laboratorios clínicos. Un 60 % de las pacientes presenta hipertensión arterial por acúmulo de 11-desoxicorticosterona que tiene acción mineralocorticoide. El perfil de esteroides en orina muestra un patrón de metabolitos del cortisol reducido y un incremento de los metabolitos de la 11-desoxicorticosterona [59].

Existen formas leves del déficit enzimático [60]. El diagnóstico molecular deberá confirmar la presencia en homocigosis o heterocigosis compuesta de mutaciones en el gen *CYP11B1* (Tabla 2).

La resistencia a los glucocorticoides es una causa muy infrecuente de virilización del feto femenino. A causa de una mutación en el gen del receptor de glucocorticoides (*GRα* o *NR3C1*) (Tabla 2) hay una hipersecreción de cortisol y de ACTH sin evidencia clínica de hipercortisolismo, pero con manifestaciones de exceso de andrógenos y de mineralocorticoides [61].

La resistencia a los estrógenos por mutaciones inactivadoras en el receptor alfa del E2 (gen *ESR1*) (Tabla 2) es una patología muy infrecuente, primero descrita en el sexo masculino; en el femenino, la morfología de los genitales externos al nacer no ha sido descrita pero desarrollan una virilización postnatal por desarrollo de un ovario poliquístico con aumento de androstendiona y T, así como talla alta y osteoporosis [62].

#### b) Aumento de andrógenos de origen fetoplacentario

La P450-oxidorreductasa (POR), es una flavoproteína unida a la membrana del citocromo C que juega un papel fundamental en la transferencia de electrones del NADPH a los enzimas microsomales P450 (CYP21, CYP17 y CYP19 o aromatasa). El déficit de POR se caracteriza por déficit parcial y muy variable de varias actividades enzimáticas (Figura 2): 17α-hidroxilasa y 17,20-desmolasa que puede o no asociarse a déficit de 21-hidroxilasa y de aromatasa. Los pacientes pueden presentar un amplio espectro fenotípico y asociarse a malformaciones esqueléticas características (síndrome de Antley-Bixler). Puede ser causa de HSC y de ambigüedad genital [63]. Desde el punto de vista bioquímico pueden presentar concentraciones normales o bajas de cortisol, elevadas de 17OH-P, eventualmente de T y concentraciones anómalas de algunos de los esteroides (y sus metabolitos) de la “vía de la puerta trasera” (Figura 3). El perfil urinario de esteroides muestra el acúmulo de metabolitos de la pregnenolona y de la P. Las niñas afectadas nacen con genitales ambiguos por exceso de andrógenos intrauterinos debido al déficit de aromatasa (CYP19) y/o por la síntesis de DHT por la vía de la puerta trasera [64]. La madre puede haber manifestado signos de virilización durante el embarazo con concentraciones elevadas de T. La virilización de la niña no progresa y las concentraciones de andrógenos circulantes no son elevadas hasta que, al llegar la pubertad, puede manifestarse un déficit de síntesis ovárica de E2 y de nuevo un aumento de precursores de la vía trasera de la esteroidogénesis [65]. El diagnóstico molecular debe confirmar la presencia en homocigosis o heterocigosis compuesta de mutaciones inactivadoras en el gen *POR* (Tabla 2)*.*

La enzima aromatasa (CYP19) cataliza el paso de T a E2 y de androstendiona a estrona (E1). En ausencia de actividad aromatasa la placenta no puede convertir el sulfato de DHEA, producido en grandes cantidades por la adrenal fetal, en estrógenos (E1, E2 y estriol) y se convierte en T lo que provoca la virilización del feto 46,XX y de la madre [66]. Suele observarse un aumento de T y de gonadotropinas, en especial de la FSH. El diagnóstico molecular debe confirmar la presencia de mutaciones en homocigosis o heterocigosis compuesta en el gen *CYP19A1* (Tabla 2)*.*

Tumores fetales o placentarios productores de andrógenos: se ha descrito algún caso de tumor suprarrenal congénito responsable de la virilización de un feto 46,XX.

#### c) Aumento de andrógenos de origen materno

El exceso de andrógenos puede proceder de la madre por presencia de tumores virilizantes durante el embarazo (como el luteoma del embarazo o el tumor de Krukenberg), por tratamiento fármacológico o por contaminantes medioambientales con efecto androgénico o incluso ser debido a un mal control terapéutico durante el embarazo de una madre, afecta también de HSC (Tabla 1).

### 3) Desarrollo anómalo de los genitales internos

Existen malformaciones aisladas de los conductos genitales internos femeninos (útero, vagina y trompas de Falopio). Pueden ser debidas a un desarrollo incompleto o a la presencia de estructuras anómalas (Tabla 1). No son frecuentes y, en algunos casos, presentan incidencia familiar, por lo que una posible causa genética puede ser invocada, aunque en escasas ocasiones llega a ser aclarada.

No existen marcadores bioquímicos específicos. Sólo la clínica de amenorrea, dismenorrea, infertilidad puede orientar el diagnóstico. Las malformaciones consisten en aplasia o hipoplasia de útero y trompas, útero bicorne o bipartito que pueden asociarse a malformaciones de otros sistemas o tejidos, como en el síndrome Pie-Mano-Genital (para el que se ha descrito un gen asociado como *HOXA13*), el síndrome MURCS (*Müllerian aplasia, Renal aplasia, Cervico-thoracic Somite abnormalities*, definido como multigénico hasta la actualidad) y el síndrome MRKH (*Mayer-Rokitansky-Küster-Hauser*) tipos I y II en el que se describen varias anomalías genéticas y finalmente las mutaciones inactivadoras en el gen *WNT4* para las que hemos descrito un posible desarrollo ovotesticular o testicular y que también pueden presentar una aplasia de conductos Müllerianos (Tabla 2).
